# Identification of candidate plasma miRNA biomarkers for the diagnosis of head and neck squamous cell carcinoma

**DOI:** 10.2144/fsoa-2023-0189

**Published:** 2024-05-20

**Authors:** Weixing Liu, Yue Liu, Pei Li, Jia Chen, Jiamin Liu, Zhi Shi, Hui Liu, Jin Ye

**Affiliations:** 1 Department of Otolaryngology, Head and Neck Surgery, Third Affiliated Hospital of Sun Yat-sen University, Guangzhou, Guangdong, China; 2 Division of Pulmonary and Critical Care, Department of Internal Medicine, Third Affiliated Hospital, Sun Yat-sen University, Guangzhou, Guangdong, China; 3 Jinan University, Guangzhou, Guangdong, China

**Keywords:** biomarker, diagnosis, head and neck cancer, miRNA, plasma

## Abstract

**Aim:** Current head and neck squamous cell carcinoma (HNSCC) diagnostic tools are limited, so this study aimed to identify diagnostic microRNA (miRNA) biomarkers from plasma. **Materials & methods:** A total of 76 HNSCC and 76 noncancerous control (NC) plasma samples underwent microarray analysis and quantitative reverse transcription PCR to screen for diagnostic plasma miRNAs. The diagnostic potential of the miRNAs was evaluated by the receiver operating characteristic curve. **Results:** miR-95-3p and miR-579-5p expression was shown to be significantly upregulated, and that of miR-1298-3p to be downregulated in HNSCC patients compared with controls. The final diagnostic panel included miR-95-3p, miR-579-5p and miR-1298-3p with an area under the curve of 0.83. **Conclusion:** This three-miRNA panel has potential for the diagnosis of HNSCC.

## Plain language summary

Early detection of head and neck cancer is crucial. In this study, we established a diagnostic model based on blood samples. This is a convenient diagnostic and screening tool that can help people early detect head and neck cancer.

## Abstract (plain language summary)

Head and neck squamous cell carcinoma is one of the most prevalent cancers worldwide, and more than 60% of new cases are diagnosed at an advanced stage.

Traditional tumor screening tools, including imaging and the analysis of protein markers, are insufficient for early detection of disease.

Liquid biopsy of miRNAs is a non-invasive technique that can be easily used to screen patients.

A total of 192 plasma miRNAs were differentially expressed between HNSCC patients and non-cancerous controls in the screening cohort, including 37 upregulated and 155 downregulated.

The top ten most significantly upregulated and downregulated miRNA target mRNAs were predicted by miRDB and TargetScan.

GO and KEGG analyses for target mRNAs were included in the bioinformatics analysis.

miR-95-3p and miR-579-5p were significantly upregulated and miR-1298-3p was downregulated in HNSCC patients in the validation set compared with non-cancerous controls.

Further analysis showed that miR-95-3p and miR-1298-3p expression was significantly correlated with the clinical stage of HNSCC.

The final diagnostic panel included miR-95-3p, miR-579-5p and miR-1298-3p, with an area under the curve of 0.83.

Head and neck cancer (HNC) is one of the most prevalent cancers worldwide, and caused approximately 460,000 deaths in 2020 [1]. The most common histological type of HNC is squamous cell carcinoma (HNSCC), which occurs primarily in the oral cavity, pharynx and larynx. Early detection is crucial to allow for prompt treatment, but unfortunately more than 60% of new HNSCC cases are diagnosed at an advanced stage, so patients have a poor prognosis [2].

Diagnosis usually requires an endoscopic examination, imaging and a tissue biopsy. However, the high cost and invasiveness of these methods limit their use as widespread screening tools for HNSCC. Therefore, there is an urgent need to develop a convenient diagnostic screening tool to help general practitioners detect and triage HNSCC patients.

In recent years, the non-invasive technique of liquid biopsy has gathered interest for the detection of HNC. This procedure assesses analytes such as cell-free RNA [3], circulating tumor cells [4], cell-free tumor DNA [5], and extracellular vesicles [6] from biological fluids including blood, saliva, and urine. Each type of biomarker provides different information on disease status.

MicroRNAs (miRNAs) are non-coding RNAs of 18–25 nucleotides in length that post-transcriptionally regulate gene expression [7]. They are extremely stable [8,9], and can be detected both in tissue samples and via minimally invasive means. Furthermore, studies have suggested their potential use as prognostic and predictive biomarkers for HNSCC development and progression as they were found to be dysregulated in tumor tissue compared with healthy tissue [^10-12^]. The release of tumor cells into the blood occurs at an early stage of HNSCC disease [13], and primary tumors or circulating tumor cells can release miRNAs directly into the plasma [3].

The current study aimed to identify plasma miRNAs that are differentially expressed between HNSCC patients and non-cancerous controls that could be used as biomarkers in the diagnosis of HNSCC.

## Materials & methods

### Collection of study specimens

Peripheral blood was obtained from 76 HNSCC patients and 76 non-malignant controls (NCs) from the Third Affiliated Hospital of Sun Yat-sen University (Guangzhou, China) between 2019 and 2022 and stored in EDTA tubes. Five patients with HNSCC and five NCs were randomized to the screening group, and 71 HNSCC patients and 71 NCs were selected for the validation set. The Ethics Committee of the Third Affiliated Hospital of Sun Yat-sen University approved this study.

### Plasma miRNA extraction

Blood samples were centrifuged at 400× *g* for 10 min, and the supernatant was collected and recentrifuged at 1000× *g* for a further 10 min before being transferred to a new Eppendorf tube and stored at -80 °C until analysis. TRIzol reagent (Invitrogen, CA, USA) was used to isolate total RNA from screening group samples for microarray analysis. The miRNeasy Serum/Plasma Advanced Kit (Qiagen, Hilden, Germany) was used to extract total RNA from 400 μl plasma samples from the validation group according to the manufacturer's instructions. Five μl of synthetic cel-miR-39 (5 nM/l, RiboBio, Guangzhou, China) was added to denatured plasma as a standard external reference. The NanoDrop ND-1000 spectrophotometer was used to determine the total amount of RNA and its purity.

### miRNA microarray analysis

The NEBNext Multiplex Small RNA Library Prep Set for Illumina (NEB, MA, USA) was used to generate sequencing libraries according to the manufacturer's instructions. miRNAs from plasma samples were then subjected to RNA sequencing using the NextSeq 500 platform, and miRDeep2 software was used to identify known and novel miRNAs [14]. Data normalization and the analysis of miRNA differential expression (DEmiRNAs) were performed using R package ‘edgeR’ [15]. Fold-changes ≥2 and p < 0.05 were considered statistically significant.

### Bioinformatics analysis

Target mRNAs of miRNAs were predicted by TargetScan (http://www.targetscan.org/vert_71/) using the miRDB database [16]. The R package ‘Cluster Profiler’ was used to perform gene ontology (GO) and Kyoto Encyclopedia of Genes and Genomes (KEGG) pathway analysis [17]. The online tool STRING (http://string.embl.de/) was used to predict the protein–protein interaction (PPI) network. A composite score larger than 0.7 was used as the cut-off criterion. Cytoscape software was used to determine the hub genes in the PPI network.

### miRNA reverse transcription & quantitative (q)PCR

The miRNA 1st strand cDNA synthesis kit (Accurate Biotechnology, Changsha, China) was used to synthesize cDNA. Real-time qPCR was performed on a 96-well plate in a Bio-Rad CFX 96 real-time detection system using the following conditions: 95 °C for 2 min, followed by 40 cycles of 95 °C for 3 s and 60 °C for 20 s. Average CT values was used to calculate ΔCT. Equation 2-ΔΔCT was used to calculate relative miRNA expression.

### Statistical analysis

SPSS (version 25) and GraphPad Prism (version 8.0.1) software were used to perform data analysis. Receiver operating characteristic curves and the area under the curve (AUC) were used to analyze the diagnostic efficacy of plasma miRNAs in the diagnosis of HNSCC. Logistic regression was used to predict the diagnostic effects of multiple biomarkers. P-values less than 0.05 were considered statistically significant.

## Results

### Screening DEmiRNAs

**Figure 1.**
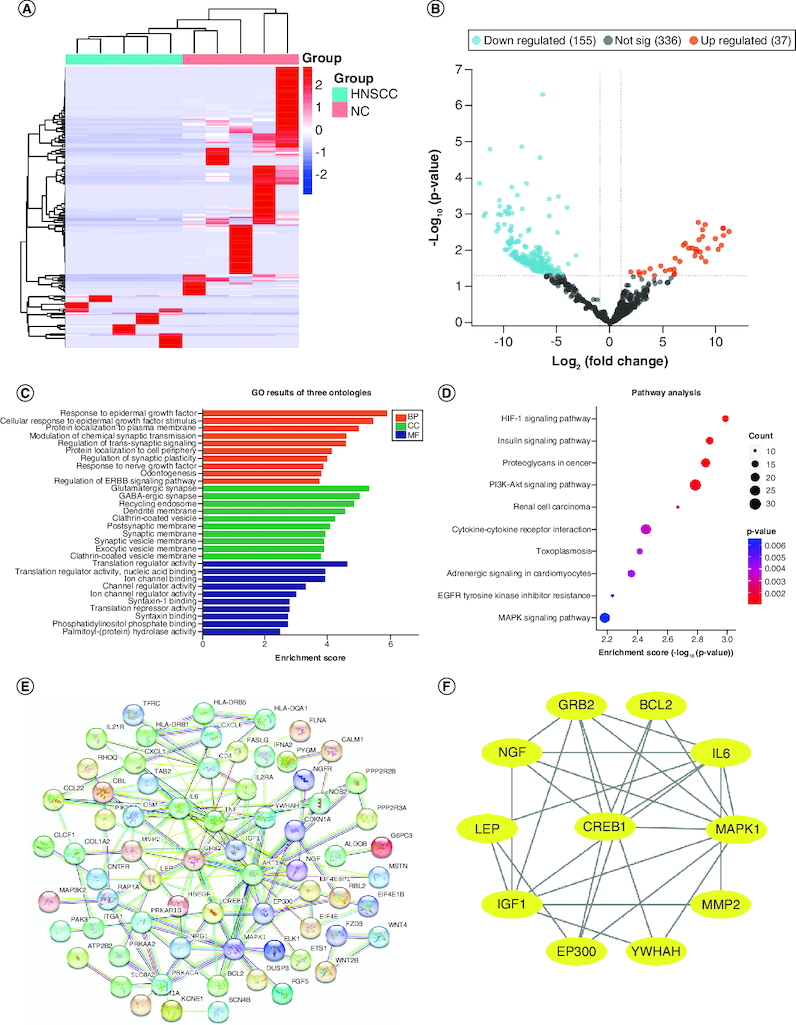
Differentially expressed plasma miRNAs in the screening cohort. **(A)** heatmap and **(B)** volcano plot demonstrating differentially expressed miRNAs between head and neck squamous cell carcinoma (HNSCC) patients and non-malignant controls (NCs). **(C)** Gene ontology analysis. **(D)** The top ten most significant KEGG pathways. **(E)** PPI network of potential target genes enriched in the top ten KEGG pathways. **(F)** The PPI network of hub genes originating from **(E)** with the most significant interactions. miRNAs with significant differential expression were defined as having a fold-change ≥2 and p < 0.05.

Clinical characteristics of the screening set are shown in Table 1, including age, sex, anatomic site, and tumor stage. Total RNA was extracted from screening set plasma samples, and a total of 1112 DEmiRNAs were identified by microarray analysis, including 587 known and 525 novel miRNAs. Among the 587 known miRNAs, 192 were differentially expressed between HNSCC and NCs samples, including 37 upregulated and 155 downregulated (Figure 1A & B).

**Table 1.** Characteristics of the screening and validation sets.

Characteristics	Screening cohort			Validation cohort		
	HNSCC	NCs	p-value	HNSCC	NCs	p-value
Total	5	5		71	71	
Gender			0.778			0.16
– Male	4	4		66	62	
– Female	1	1		5	9	
Age	53–61	41–63	0.25	35–79	36–74	0.29
Anatomic site						
– Oral cavity	0			6		
– Oropharynx	0			4		
– Larynx	4			15		
Hypopharynx	1			46		

### Bioinformatics analysis

The top ten significantly upregulated and downregulated miRNA target mRNAs were predicted by miRDB and TargetScan. GO and KEGG analyses of these target mRNAs were undertaken to explore the function of DEmiRNAs (Figure 1C & D). GO enrichment showed that ‘response to epidermal growth factor’, ‘glutamatergic synapse’ and ‘translation regulator activity’ were the most common biological processes among biological processes, cellular components, and molecular functions, respectively. KEGG pathway analysis suggested that DEmiRNAs were most enriched in the HIF-1 signaling pathway (Figure 1E). DEmiRNA target genes enriched in the top ten KEGG pathways were used to construct the PPI network, and hub genes were identified as *EP300*, *GRB2*, *IGF1*, *BCL2*, *LEP*, *MAPK1*, *IL6*, *MMP2*, *CREB1*, *YWHAH* and *NGF* (Figure 1F).

### Validation of candidate miRNAs

**Figure 2.**
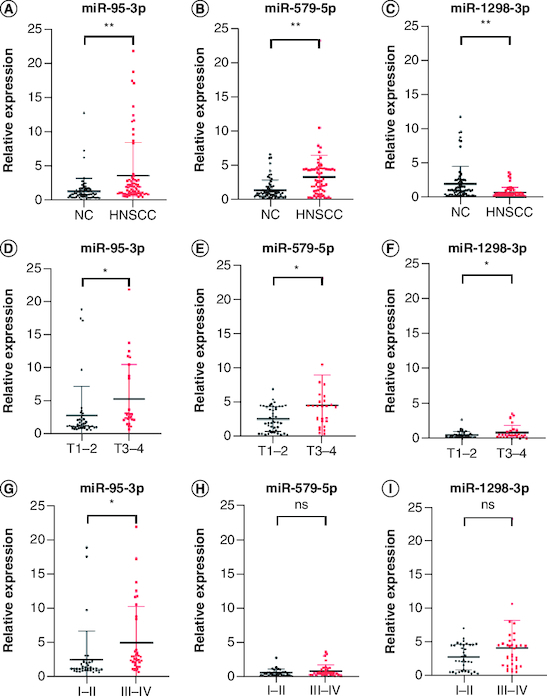
Plasma expression levels of miR-95-3p, miR-579-5p, and miR-1298-3p in the validation set. The expression of **(A)** miR-95-3p, **(B)** miR-579-5p, and **(C)** miR-1298-3p in plasma was differentially expressed in HNSCC patients compared with NCs. The expression of **(D)** miR-95-3p, **(E)** miR-579-5p, and **(F)** miR-1298-3p in T1–T2 compared with T3–T4 disease. **(G)** miR-95-3p, **(H)** miR-579-5p, and **(I)** miR-1298-3p expression in stage I–II versus stage III–IV disease. **p < 0.01; *p < 0.05. ns: Not significant.

The available clinical characteristics of the validation cohort are summarized in Table 1. Among the top ten significantly up- and downregulated DEmiRNAs, 14 had a CT <35, suggesting that they could be reliably measured in plasma. Validation analysis of these 14 miRNAs found that miR-95-3p and miR-579-5p were significantly upregulated and miR-1298-3p was downregulated in HNSCC patients compared with controls (Figure 2A–C). Further analysis showed that the T stage and tumor stage was significantly correlated with the expression of miR-95-3p and miR-1298-3p (Figure 2D, F, G & I), but not of miR-579-5p (Figure 2E & H). The following primers were obtained from Sangon Biotech: miR-95-3p forward, 5′-CACGCTTCAACGGGTATTTATTG-3′; miR-579-5p forward, 5′-AACAATTCGCGGTTTGTGCC-3′; and miR-1298-3p forward, 5′-ACAAGCATCTGGGCAACTGA-3′.

### Design of the diagnostic model

**Figure 3.**
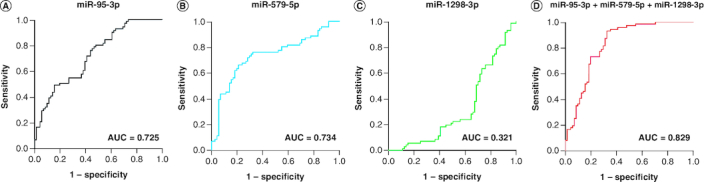
Receiver operating characteristics curve of miR-95-3p, miR-579-5p, miR-1298-3p and their combination to differentiate HNSCC patients from non-malignant controls. **(A)** miR-95-3p. **(B)** miR-579-5p. **(C)** miR-1298-3p. **(D)** The combination of miR-95-3p, miR-579-5p and miR-1298-3p.

miR-95-3p and miR-579-5p showed a high diagnostic efficiency for HNSCC patients in the validation group, with AUC values of 0.725 and 0.734 (Figure 3A & B), respectively. The AUC of miR-1298-3p was 0.321 (Figure 3C). Logistic regression was used to establish the optimal panel for HNSCC detection, which consisted of miR-95-3p, miR-579-5p and miR-1298-3p, with the following formula:

Logit(P)=0.169 × miR-95-3p+0.075 × miR-579-5p - 0.101 × miR-1298-3p

The AUC of the three-miRNA panel was 0.83 (Figure 3D), indicating a high diagnostic precision for patients with HNSCC.

## Discussion

Approximately 700,000 new cases of HNSCC are diagnosed each year [1]. Current treatments include surgery, radiochemotherapy and immunotherapy, but patient prognosis remains poor [18]. The most economical and effective means of improving prognosis is early detection of disease, but traditional tumor screening tools, including imaging and protein markers, are insufficient. Liquid biopsy of miRNA is a non-invasive technique that can easily be used to screen patients. Circulating miRNAs are relatively stable in plasma or serum, and play a critical role in tumor cell communication [19]. Therefore, they have the potential to be used as candidate biomarkers for the early diagnosis of tumors.

In this study, we identified the top ten significantly up- and downregulated DEmiRNAs in HNSCC plasma, which were shown to play important roles in the formation and development of HNSCC. HIF-1 signalling was the top hit in KEGG pathway analysis, and has been implicated in several human cancers [20]. Moreover, the hub gene with the highest score in the PPI network, IL-6, was previously found to be associated with hypoxia-induced activation of MAPK, HIF-1α and NF-κB [21]. Therefore, it is plausible that plasma miRNAs affect mRNA expression in HNSCC.

We also showed that miR-95-3p and miR-579-5p were significantly upregulated in the plasma of patients with HNSCC compared with controls, while miR-1298-3p was downregulated. miR-95-3p and miR-579-5p have the potential to be good plasma biomarkers for HNSCC, with AUC values of 0.725 and 0.734, respectively, although their diagnostic accuracy was improved in combination with miR-1298-3p, which increased the AUC value to 0.83.

miR-95-3P was previously reported as a diagnostic and prognostic marker for osteosarcoma [22], and is also upregulated in cervical carcinoma where it promotes tumor development by regulating VCAM-1 [23]. Furthermore, miR-95-3p is upregulated in prostate cancer tissues and correlates with poor prognosis [24], while it also affects the progression of triple-negative breast cancer by targeting AKAP12 expression [25].

miR-579-5P is a downstream effector of small nucleolar RNA host gene 25, which negatively impacts on MAPK signaling [26]. High expression of hsa-miR-579-5p was previously shown to be associated with short survival time in hepatocellular carcinoma patients [27], and exosomal miR-579-5p was reported to be a hypoxia-related biomarker in prostate cancer [28]. Finally, miR-1298-3p exerts an inhibitory effect in glioma cells by downregulating nidogen-1 [29], and has a similar negative regulatory role in cervical cancer cells [30].

Plasma miRNAs in oral squamous cell carcinoma have previously been studied, with Pedersen *et al.* identifying miR-30a-5p and miR-769-5p as potential biomarkers for oral cancer diagnosis and recurrence [31]. Although plasma miRNAs can serve as predictors of cisplatin-induced nephrotoxicity in HNC patients [32], and aberrantly expressed miRNAs were reported to be potential diagnostic biomarkers for HNSCC [33,34], the functional roles of miR-95-3p, miR-579-5p and miR-1298-3p in the head and neck are unclear. Therefore, further investigation of this is needed, and multicenter studies should also be undertaken to confirm the current findings.

A limitation of this study was the small sample size of the screening group, but we used a large validation group sample to validate the top ten significantly up- and downregulated DE miRNAs. Plasma miRNAs have several advantages as diagnostic or prognostic biomarkers. First, the stability of extracellular circulating miRNAs facilitates the measurement of miRNAs from patients for whom sample storage may be necessary. Second, plasma protects against miRNA loss from coagulation [35]. Third, changes in the abundance of plasma miRNAs after radiochemotherapy can be used as a novel biomarker to monitor therapy [36,37]. Furthermore, serum miRNAs can be measured routinely and efficiently. However, the lack of standardized protocols and methodologies is a major limitation [38], so this is needed to improve the biomarker potential of miRNAs.

## Conclusion

Plasma levels of miR-95-3p, miR-579-5p, and miR-1298-3p were found to be differentially expressed in HNSCC patients compared with NCs, so were identified as promising biomarkers for the diagnosis of HNSCC.
